# RASS: a web server for RNA alignment in the joint sequence-structure space

**DOI:** 10.1093/nar/gku429

**Published:** 2014-05-15

**Authors:** Gewen He, Albert Steppi, Jose Laborde, Anuj Srivastava, Peixiang Zhao, Jinfeng Zhang

**Affiliations:** 1 Department of Computer Science, Florida State University, Tallahassee, FL 32306, USA; 2 Department of Statistics, Florida State University, Tallahassee, FL 32306, USA

## Abstract

Comparison of ribonucleic acid (RNA) molecules is important for revealing their evolutionary relationships, predicting their functions and predicting their structures. Many methods have been developed for comparing RNAs using either sequence or three-dimensional (3D) structure (backbone geometry) information. Sequences and 3D structures contain non-overlapping sets of information that both determine RNA functions. When comparing RNA 3D structures, both types of information need to be taken into account. However, few methods compare RNA structures using both sequence and 3D structure information. Recently, we have developed a new method based on elastic shape analysis (ESA) that compares RNA molecules by combining both sequence and 3D structure information. ESA treats RNA structures as 3D curves with sequence information encoded on additional coordinates so that the alignment can be performed in the joint sequence-structure space. The similarity between two RNA molecules is quantified by a formal distance, geodesic distance. In this study, we implement a web server for the method, called RASS, to make it publicly available to research community. The web server is located at http://cloud.stat.fsu.edu/RASS/.

## INTRODUCTION

Comparison of ribonucleic acid (RNA) structures is an effective tool for studying the functions of RNA molecules and their evolutionary relationships. There have been a number of methods developed for RNA structure alignment/comparison (1–15). Databases for RNA structures have also been built to facilitate easy retrieval of such data (14,16,17). There are mainly three types of structure comparison methods. The first type of methods focuses on detection of local structural motifs to identify functional domains, such as NASSAM (9), COMPADRES (8), RNAMotifScan (11) and FR3D (7). The second type of methods reduces the three-dimensional (3D) structures to one-dimensional (1D) sequences by representing nucleotide residues with some local structure features. Existing sequence alignment methods can then be applied to align the resulting 1D sequences. Among this type of methods, iPARTS discretized backbone torsion angles to form structural alphabet with 23 letters (states). A substitution matrix is then derived for the 23 states and used in the sequence alignment (2). SARA uses a set of unit vectors derived from consecutive nucleotides to represent each nucleotide, which can be compared with other nucleotide using unit-vector root mean square (URMS) as distance (1,18). LaJolla uses an n-gram model to analyze sequences derived from nucleotide torsion angles (4). Similarly, PRIMOS/AMIGOS (5) and DIAL (6) also represent nucleotides with torsion angles and align the sequences encoded by the torsion angle representation. These methods do not necessarily produce globally similar alignment between two RNA structures (i.e. with small RMSDs (root-mean-square-deviations)). To minimize RMSD for the aligned parts between two RNA structures, extra steps are required after the sequence alignment. The third type of methods starts from aligning similar local structures and then obtains larger scale alignment by extending the initial local alignment. For example, R3D Align employs a maximum clique algorithm on a specially defined graph, called a local alignment graph, to merge local alignments to form a global alignment (10); ARTS uses P (phosphor) atoms of two consecutive base pairs as seeds to first find structurally similar seed quadrants and then aligns overall structures based on the alignment of the seed quadrants (3); SETTER decomposes RNA structures to larger local structure units, called generalized secondary structure units (GSSUs), and uses a pairwise comparison method based on 3D similarity of the GSSUs (15). Several web servers for RNA structure alignment have been developed implementing some of the above methods, including ARTS (3,19), SARA (1,18), SETTER (15,20), RADAR (21) and SARSA (22). 

Sequence alignment and structure alignment use different sets of information: the former uses information of side chains, which are reduced to single letters, and the latter uses information of the backbone geometry. Both types of information play important roles in determining RNA functions. In principle, structure alignment methods should utilize sequence information since such information is almost always available. Recently, we have developed a RNA structure alignment method (23,24) that aligns RNAs in the joint sequence-structure space using a framework based on elastic shape analysis (ESA) originally designed for protein structure comparison (25,26). We have shown that the method performed better than using either sequence or structure information alone. The method also performed better than previous methods in RNA function prediction when tested on a benchmark dataset. In this study, we develop a web server for joint sequence-structure comparison of RNA structures using ESA, called RASS (RNA alignment in the joint sequence-structure space). In the next section, we first briefly describe the methodology of RASS, which is followed by a detailed description of the usage of the web server.

## MATERIALS AND METHODS

We use ESA to compare two RNA structures or structural fragments. ESA treats RNA structures as 3D curves and utilizes a geometric framework that has been developed originally in image analysis and computer vision for shape analysis of parameterized curves and surfaces (27–30). The basic idea in this framework is to design an infinite-dimensional topological space (manifold) of curves, endow it with a metric structure, and compare any two objects by computing the distances between them on this manifold. Under this framework, we will be able to quantify the similarity between any two RNA structures by a formal distance, geodesic distance, computed on their respective shape manifolds. A geodesic path, the shortest path connecting the two curves (two points in the manifold), can also be generated. It can be seen as an optimal deformation from one structure into the other. The framework allows us to seamlessly incorporate sequence information into 3D structures in the computation of geodesic distances. The detailed description of the ESA method is given in (23) and (25).

The implementation is done using Matlab (with some embedded C functions). Due to Matlab initialization, RNA structure comparison at the web server takes more time than running the programs locally.

## WEB INTERFACE AND USAGE

### Input

**Figure 1.**
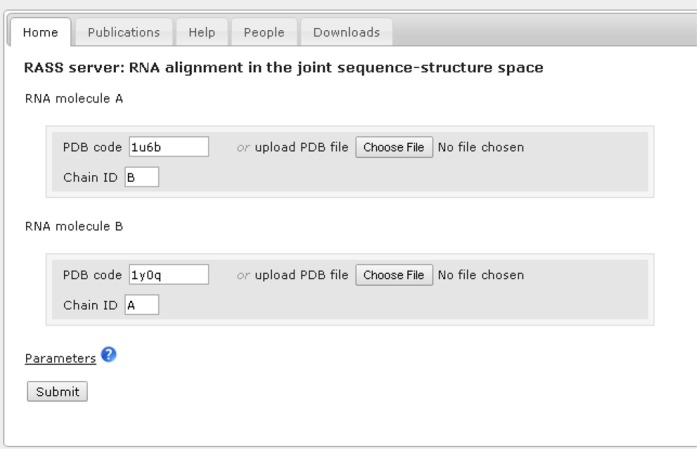
The user interface of RASS.

The user interface of RASS is shown in Figure 1. The input of the server takes two RNA chains (structure A and B). Users can provide a Protein Data Bank (PDB) code or upload a PDB file for each RNA structure. A chain name should also be provided for each RNA structure.

### Parameter selection

The parameters are preset at default values. Users have the flexibility to specify values for the following:

- Starting (for each structure A and B): the index of the starting residue from the RNA chains. The default value is 1.
- Ending (for each structure A and B): the index of the ending residue from the RNA chains. The default value is 10 000. That means for RNA chains with more than 10 000 residues, without specifying the starting and ending positions of the residues, the first 10 000 residues will be used in comparison. If the length of the RNA chain is less than 10 000, then the actual chain end position will be used. This value is large enough for the RNA structures currently in PDB. Being able to select starting and ending positions from the RNA chains to be compared is a feature most of the existing web servers do not provide. With this option users can select a partial structure within a larger RNA molecule for comparison, which can be interesting either structurally or functionally. Since our method is a global alignment method, this option can be especially useful if a user wants to compare a partial structure based on some prior knowledge/information.
- Lambda: weight for the nucleotide sequence (23). The value of lambda needs to be greater or equal to zero. When zero is specified, the resulting geodesic distance takes into account only backbone geometry information but not the nucleotide sequences. When a large number (e.g. 70) is specified, then the distance computation is dominated mainly by nucleotide sequences. Recommended values for lambda is between 0 and 10. The optimal value for lambda obtained by cross-validation on a benchmark data is 5, which is set as the default value.

### Output

**Figure 2.**
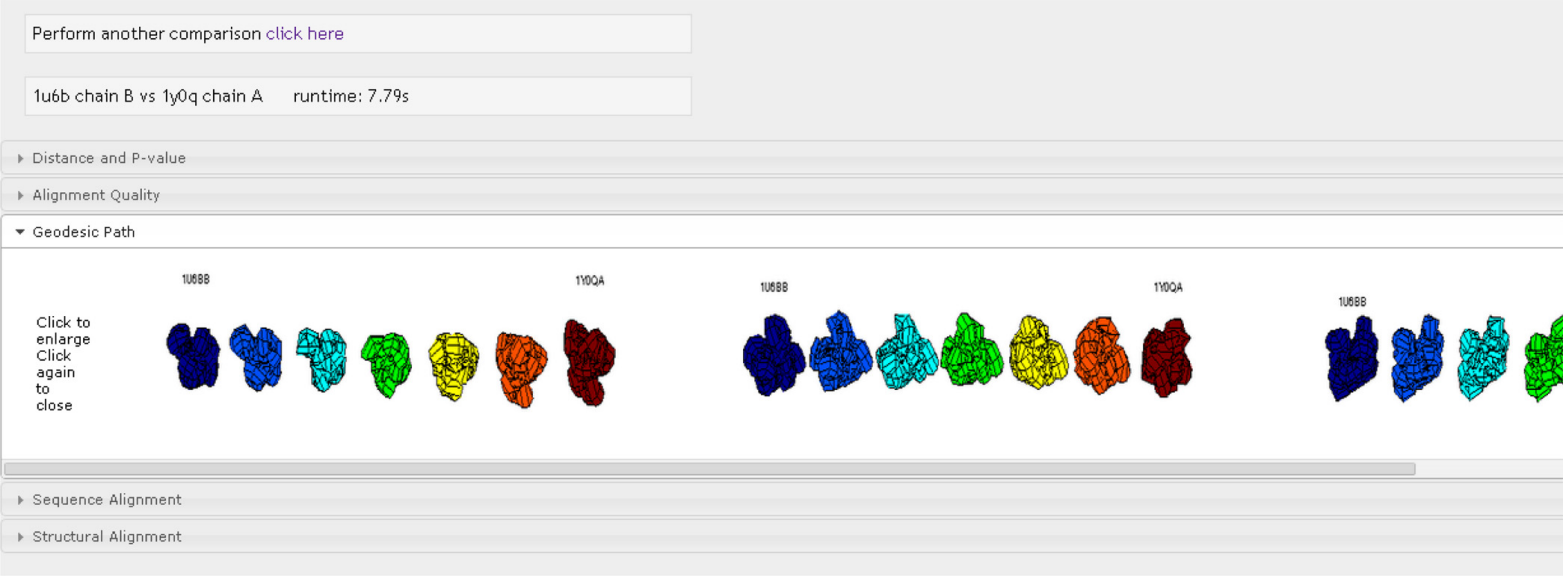
Geodesic path for chain B of RNA 1u6b and chain A of RNA 1y0q.

**Figure 3.**
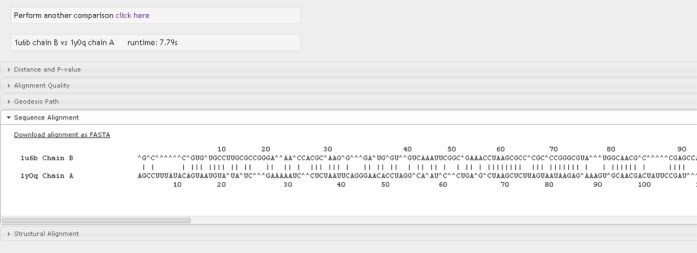
Sequence alignment obtained from global matching of two RNA molecules.

**Figure 4.**
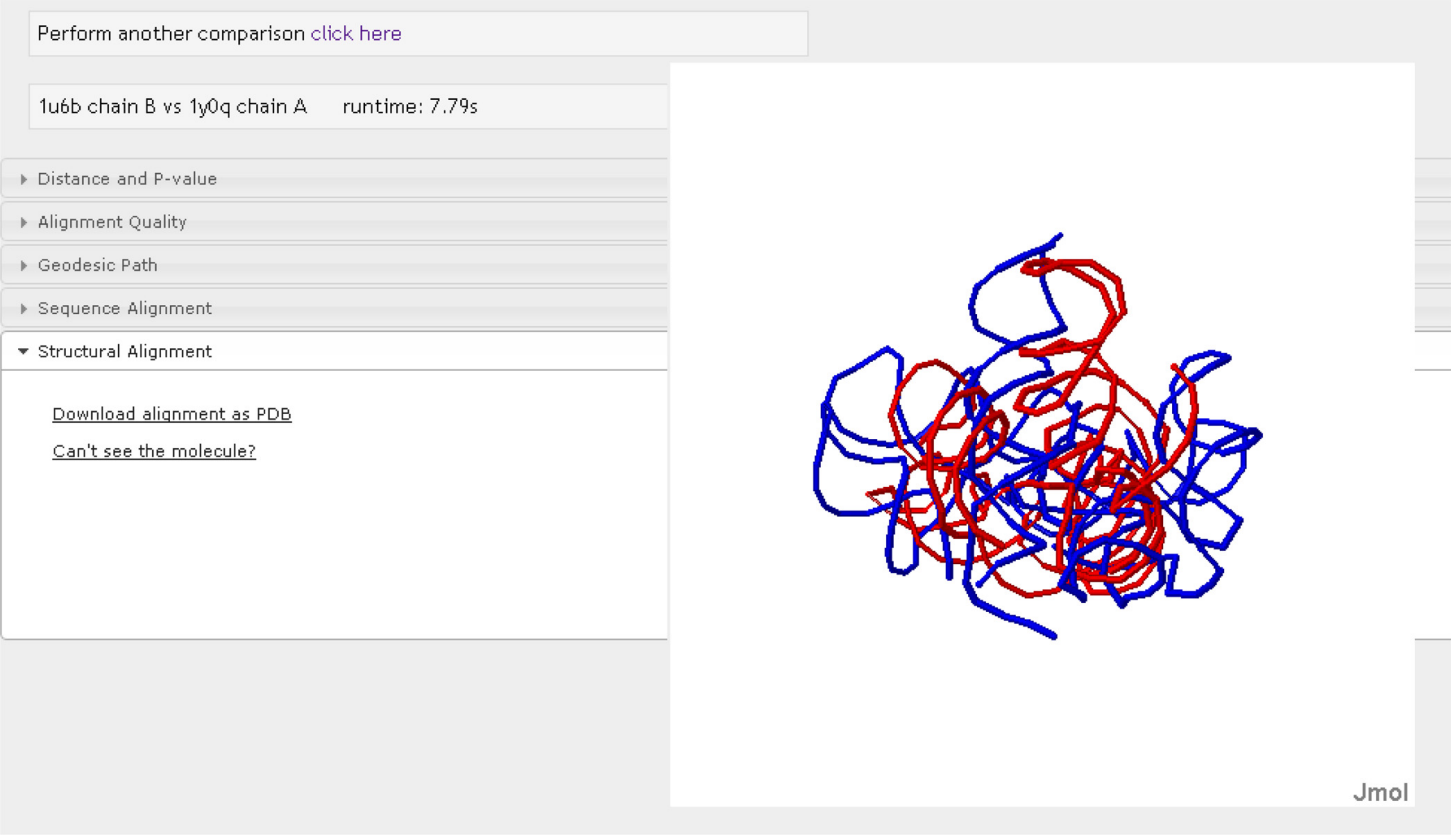
Structural superposition of the two RNA molecules displayed using Jmol.

The output is displayed in a series of drop-down tabs (see Figures 2–4):

- Distance and *P*-value: geodesic distance calculated by ESA between two RNA chains is displayed under the Distance and *P*-value tab. *P*-value is obtained by comparing the geodesic distance with an empirical Gaussian distribution derived from a set of pairwise distances computed using a large number of non-homologous RNA structures taken from PDB (31). A discussion on the distribution of geodesic distances is given in Supplementary material. A small *P*-value indicates that the chains are related/similar statistically in the joint sequence-structure space.
- Geodesic path: this tab displays the optimal structural deformation from RNA molecule A to RNA molecule B viewed from three different angles (Figure 2). These views are slightly distorted by the sequence weight. To view an undistorted version of the geodesic path, one can set lambda = 0 (i.e. use structure only for the comparison).
- Sequence alignment: this tab shows the global sequence alignment between two RNA molecules (Figure 3). The residue indices for each molecule are shown above or below the corresponding sequence for every 10 residues.
- Structural Alignment: the 3D optimal superposition is displayed with Jmol (Figure 4). Users can download the alignment files in PDB format through the link provided in this tab. Some users may not be able to see the graphical display for the first time using the web server. A link to Jmol tutorial page is also provided.

## CONCLUSION

In this study, a web server is implemented to provide a tool for users to compare and align two RNA molecules in the joint sequence-structure space. A typical scenario for a user when using our server is as follows: The user will provide two RNA molecules as input where one may be a RNA molecule with known function and the other is a RNA molecule the user may want to know more about. After alignment of the two molecules, the user can look at the structure alignment using Jmol to identify the structural similar regions and dissimilar regions to infer how the two molecules may share similar function while differ in some substrate specificities. From the sequence alignment, the user can identify conserved nucleotides. Here, those nucleotides that align well in sequence space are also spatially close on structure space since RASS aligns both sequence and structure simultaneously. From the aligned nucleotides, the user can gain more insight on the functional and/or evolutionary relationship of the two molecules.

For pairwise alignment of a large set of RNA structures, users can download the freely available programs provided at http://stat.fsu.edu/∼jinfeng/ESA.html.

## SUPPLEMENTARY DATA

Supplementary Data are available at NAR Online.

Supplementary Data
