# Supplementary material for: RASS: a web server for RNA alignment in the joint sequence-structure space
Source: Nucleic Acids Res. 2014 May 15;42(Web Server issue):W377–81. doi: 10.1093/nar/gku429 (PMC4086137; doi:10.1093/nar/gku429)
Supplement: Supplementary Data [file supp_42_W1_W377__index.html]

Supplementary Data 

# RASS: a web server for RNA alignment in the joint sequence-structure space

## Supplementary Data

**Files in this Data Supplement:**

- SUPPLEMENTARY DATA
