# Supplementary material for: RASS: a web server for RNA alignment in the joint sequence-structure space
Source: Nucleic Acids Res. 2014 May 15;42(Web Server issue):W377–81. doi: 10.1093/nar/gku429 (PMC4086137; doi:10.1093/nar/gku429)
Supplement: Supplementary Data [file supp_gku429_nar-00287-web-b-2014-File002.docx]

Supplementary material for

RASS: A web server for RNA alignment in the joint sequence-structure space

Gewen He^1^, Albert Steppi^2^, Jose Laborde^2^, Anuj Srivastava^2^, Peixiang Zhao^1^ and Jinfeng Zhang^2,^*

^1^ Department of Computer Science, Florida State University, Tallahassee, FL, 32306, USA

^2^ Department of Statistics, Florida State University, Tallahassee, FL, 32306, USA

* To whom correspondence should be addressed. Tel: 1-850-644-3218; Fax: (850) 644-5271; Email: Jinfeng@stat.fsu.edu

Present Address: Jose Laborde, Department of Mathematics, Florida Atlantic University, Boca Raton, FL, 33431, USA

**Distribution of geodesic distances**

Figure S1 shows the distribution of geodesic distances for RNA structures in FSCOR dataset. We can see that there are two modes, one corresponding to the structures that share significant similarity in their shape and the other corresponding to structures that do not. We used Expectation and Maximization (EM) algorithm and fitted two Gaussian distributions to the data. Gaussian distributions fit the two modes reasonably well. To compute p-values, we use the Gaussian distribution corresponding to the mode where distances are from structures with significantly different shapes (one-sided test). The mode to the left is likely an artifact of the FSCOR dataset (similar structures are more enriched in FSCOR dataset). Figure S2 shows the distribution of 5055 non-homologous proteins from SCOP database. Here the main peak at around 1 corresponds to structures with different shapes. This peak is remarkably similar to the one for RNA structures in Figure S1. The peak corresponding to similar structures almost disappeared.


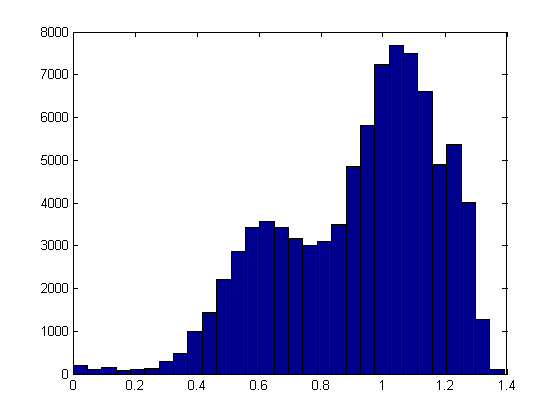


Figure S1. Distribution of pairwise geodesic distances for RNA structures in FSCOR dataset.


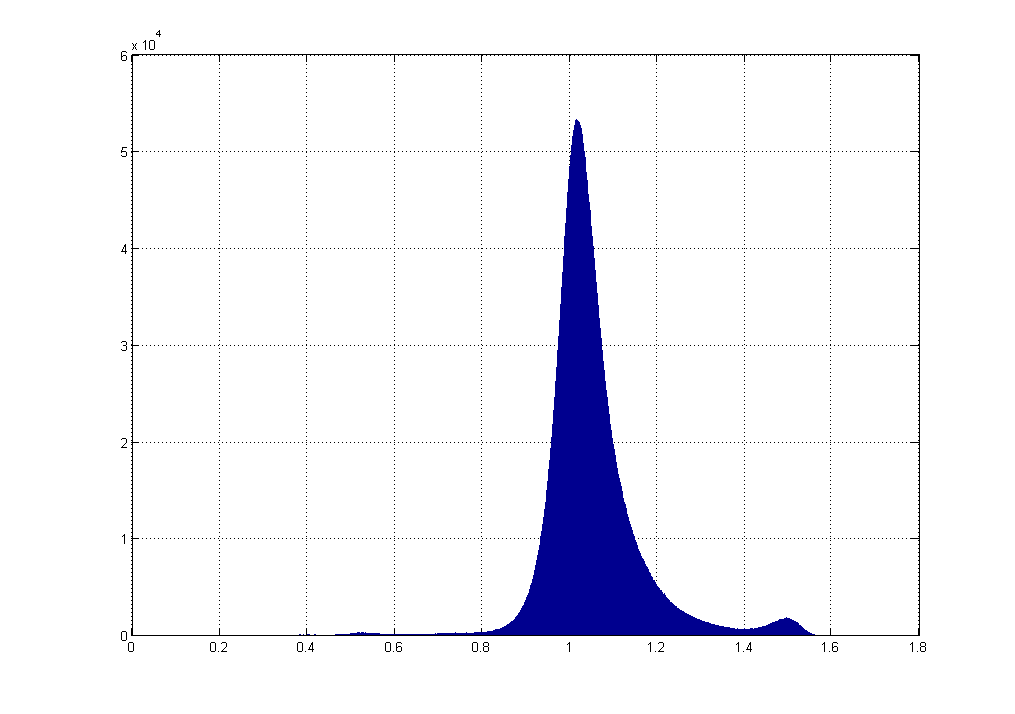


Figure S2. Distribution of pairwise geodesic distances from 5055 protein chains from SCOP database.
